# The contribution of Australian residential early parenting centres to comprehensive mental health care for mothers of infants: evidence from a prospective study

**DOI:** 10.1186/1752-4458-4-6

**Published:** 2010-04-11

**Authors:** Heather J Rowe, Jane RW Fisher

**Affiliations:** 1 Centre for Women's Health Gender and Society, Melbourne School of Population Health, Faculty of Medicine Dentistry and Health Sciences, University of Melbourne, Victoria 3010 Australia

## Abstract

**Background:**

Australia's public access residential early parenting services provide programs to assist parents who self-refer, to care for their infants and young children. Treatment programs target infant feeding and sleeping difficulties and maternal mental health. There is limited systematic evidence of maternal and infant mental health, psychosocial circumstances or presenting problems, or the effectiveness of the programs. The aim of this study was to contribute to the evidence base about residential early parenting services.

**Methods:**

A prospective cohort design was used. A consecutive sample of mothers with infants under one year old recruited during admission to a public access residential early parenting service for a 4 or 5 night stay in Melbourne, Australia was recruited. They completed structured self-report questionnaires, incorporating standardised measures of infant behaviour and maternal mood, during admission and at one and six months after discharge. Changes in infant behaviour and maternal psychological functioning after discharge were observed.

**Results:**

79 women completed the first questionnaire during admission, and 58 provided complete data. Women admitted to the residential program have poor physical and mental health, limited family support, and infants with substantial behaviour difficulties. One month after discharge significant improvements in infant behaviour and maternal psychological functioning were observed (mean (SD) daily crying and fussing during admission = 101.02 (100.8) minutes reduced to 37.7 (55.2) at one month post discharge, p < 0.001; mean (SD) Edinburgh Postnatal Depression Scale at admission = 11.3 (5.7) reduced to 6.78 (4.44), at one month, p < 0.001) which were sustained at six months. Participant satisfaction with the program was high; 58 (88%) found the support of the nurses and 50 (75%) the social support of other mothers very helpful.

**Conclusions:**

This psycho-educational approach is an effective and acceptable early intervention for parenting difficulties and maternal mood disturbance, and contributes to a system of comprehensive mental health care for mothers of infants.

## Background

Australia's residential early parenting services appear to be unique. They provide services to assist parents who are experiencing difficulties in caring for their infants and young children. There are no equivalent services in the United Kingdom, Canada, Europe or the United States of America, where these parenting needs are predominantly addressed in primary medical care, community health nursing and outreach home visiting services. In the Australian State of Victoria, there are two private and three public residential early parenting services. The private services are in hospital settings, operate on a fee for service basis subsidised by private health insurance purchased by individual families, and women are referred by their primary or specialist care physician. The public access services are supported financially by the state, free-standing in the community, and there is no cost to individual women and their families who self-refer, or are referred by health and allied health professionals. These residential early parenting services are not designated psychiatric services.

Tweddle Hospital for Babies and School of Mothercraft, a training centre for baby health care nurses was established in 1920. Since then it has adapted the nature and extent of its services in response to social change. Residential, day stay and other outreach and support programs are now offered by Tweddle Child and Family Health Service (TCFHS). Telephone triage to individual programs is conducted and there is a waiting list for the residential service of approximately 10 weeks. Records held by the service show that client satisfaction with the care provided by the service is high. The service has developed over a prolonged period and, while they have very substantial clinical expertise, there is currently only limited systematic evidence of the nature of presenting problems in mothers and infants, or of the effectiveness of these non-psychiatric programs in alleviating parent distress, promoting optimal parent-child relationships, and contributing to a comprehensive maternal and child mental health care system [1].

The aims of the present study were to document the mental health and social circumstances of women at admission to the residential program with infants aged up to twelve months, and to examine the impact of the program on maternal mental health and infant behaviour disturbance at one and six months after discharge.

## Methods

### Setting

The residential program at TCFHS is in metropolitan Melbourne, in the state of Victoria, Australia. Approval to conduct the study was given by the University of Melbourne Human Research Ethics Committee and the TCFHS Board.

### The residential program

The residential program admits up to nine families with children up to 4 years old for two, three or four night stays. The service cares for approximately 670 families per year. Each family is accommodated together in a two-room suite with a bathroom. Shared living and eating spaces provide opportunities for social interaction amongst families. Fathers are encouraged to attend and participate in the program. The program focuses on both infant and parent needs. On admission, individualised care plans are devised based on a comprehensive admission assessment.

The residential program is staffed by a team of maternal and child health nurses and early childhood professionals. The program provides support, education and role-modelling in individual and group contexts. The aim is to make parenting enjoyable for parents, to increase confidence and develop safe, effective child rearing practices. Parents work towards achieving their goals through one-to-one interaction with staff, group education sessions, self-directed learning and supported practice [2].

Group psycho-educational sessions foster understanding of infant development, including needs for sleep and play, strategies for soothing and comforting and promoting sustainable sleep habits. Facilitated discussion encourages reflection on adjustment to parenthood and includes active promotion of the important role of fathers or other supportive adults in healthy infant development and family functioning. The programs help mothers to rest, build on parents' existing strengths, educate parents about infant behaviour management strategies, assist with the development of infant care-taking and problem-solving skills, and foster parenting confidence.

The program has four phases: exploration, confidence building, skill consolidation and preparation for home. Parents learn to recognise their infant's behavioural signs of tiredness and techniques for soothing and relaxing, and practise sustainable methods for putting the baby to sleep and managing sleep disruptions [3]. Parents are assisted to establish a pattern of "feed-play-sleep" to suit their baby's individual needs. Residential staff work individually with parents to build confidence by observing and providing feedback and encouragement to assist skills development. Discharge planning includes arrangements for appropriate primary and specialist follow-up care.

The service has developed over the decades since its inception in response to community needs. Clinical practice in the service was well established prior to the widespread application of a model of evidence based practice. The theoretical foundation of the program, whilst not clearly articulated by the service, rests on the premise that assisting parents to improve their infant's behavioural regulation will diminish fatigue in infants as well as parents, and promote parenting confidence. This is intended to lead to increased parental reward and gratification from infant care-taking activities and to longer-term improvements in parent-infant attachment.

### Study design

A prospective cohort design was used with assessments made during admission to the residential program and at one and six months after discharge. Outcomes were compared with other published treatment interventions using the same psychometric measures.

### Participants

All women who were admitted to a residential program at TCHFS with a baby under one year of age with sufficient English language to complete questionnaires and standardised self-report measures were eligible and invited to participate.

### Procedures

On the first day of admission a researcher described the project orally to each woman and gave her an envelope which contained the plain language statement, the first questionnaire and a standard consent form. Participants completed the structured questionnaire and returned it with the signed consent form in a sealed envelope to a locked box during their admission (Time 1). At approximately one month (Time 2) and six months (Time 3) after discharge from the service, telephone contact was made with participants to confirm contact details and agreement to continued participation. Up to 4 attempts to make contact with participants were made if necessary, and those agreeing to continue to participate were mailed questionnaires. Completed questionnaires were returned to researchers in reply paid envelopes.

### Measurements

Three questionnaires were designed in consultation with senior staff at the service to assess known risk factors for poor postpartum mental health [4], current mental health and infant behaviour, using a combination of study specific questions and standardised, published psychometric measures. The Time 1 questionnaire addressed sociodemographic factors, psychiatric history, reproductive history including the most recent pregnancy, exposure to childhood abuse and recent violence, current physical health, coincidental adverse life events, adequacy of family support, quality of intimate partner relationship, personality style, infant behaviour and current mental health status. The postal questionnaires (Times 2 and 3) assessed: maternal health, infant behaviour, and, in order to meet US Centres for Disease Control guidelines for violence research [5], repeat assessments of fear of partner or violence. Questions about perceptions of the effectiveness of each of the components of the residential program were included in the Time 2 questionnaire. Self-rated maternal confidence in infant care was assessed in a single question at each assessment. Published, standardised psychometric measures were used to assess intimate partner relationship, infant behaviour, maternal personality style and mood. All standardised measures were completed at Time 1; mental health and infant behaviour instruments were re-administered at Times 2 and 3.

#### Sociodemographic factors

Respondents selected from a list the highest level of education they had achieved and described their main occupation prior to having a baby, which was coded according to the Australian Standard Classification of Occupations (ASCO) [6]. A question about current paid employment assessed whether participants were in full-or part-time employment in addition to their work as a mother.

#### Psychiatric history

Personal psychiatric history was defined as: self-reported past diagnosis of depression, alcohol or drug dependence, eating disorder or other serious mental illness.

#### Reproductive history

This was assessed as: experience of perinatal loss including ectopic pregnancy, termination of pregnancy for fetal abnormality, birth of a baby with congenital abnormality or of stillbirth in a prior pregnancy, and of unplanned or unwanted pregnancy, assisted conception or emergency caesarean section in the most recent pregnancy.

#### Exposure to childhood abuse

Two items assessed physical and sexual abuse from a family member, a step-parent or a person in authority in childhood. The first asked about being hit, punched, beaten or otherwise mistreated, and the second about exposure to sexual contact.

#### Exposure to violence

This was assessed as: the woman being "hit, slapped, pushed, kicked or otherwise physically hurt by someone within the last year" and whether in the past year she had been frightened of her partner. In follow-up questionnaires the same questions were asked about in the time period since the last assessment.

#### Physical health problems

Respondents endorsed items on a list of health problems: backache, headache, haemorrhoids, urinary incontinence, fecal incontinence, breast infection, unhealed or infected tear, episiotomy or caesarean wound, or other problem, described by the respondent.

#### Coincidental adverse life events

A single question asked participants about whether there are "other events in your life now that are worrying or distressing" and if so, to describe them. Responses to the open ended question were later grouped into categories.

#### Family support

A single question asked about the perceived adequacy of a woman's practical and emotional support since coming home from hospital with the current baby. Other questions assessed the availability and perceived attitudes of extended family members.

#### Intimate partner relationship

*Intimate Bonds Measure *(IBM) [7] is a 24-item assessment of quality of relationship with intimate partner yielding two independent subscale scores. The Care subscale (Chronbach's alpha = 0.94) reflects perceived sensitivity, warmth, emotional responsiveness, trust, physical gentleness and capacity for companionship. The Control subscale reflects perceived coerciveness, exertion of power and extent of criticism (Chronbach's alpha = 0.89). Additional questions assessed participant's capacity to confide in their partner, the level of partner's practical and emotional support and sexual relationship.

#### Personality style

*Vulnerable Personality Style Questionnaire *(VPSQ) [8] is a brief measure of personality traits which may increase the likelihood of developing psychological morbidity [9], which yields the Vulnerability subscale, describing over-sensitivity to the opinions of others and lack of assertiveness (Chronbach's apha = 0.77; test-retest reliability = 0.82) [8].

#### Infant health and behaviour

Study specific questions recorded details of infant health and behaviour including method of feeding, feeding difficulties, prescribed treatments for "colic", "reflux" and average length of crying episodes; the average number of times the infant had woken between 10 pm and 6 am over the preceding week, the average amount of daytime sleep, and maternal perception of overall quality of infant sleep. A record of infant behaviours was collected using the *Barr Chart *[10], a parent-completed record of duration of episodes of crying, fussing, sleeping, and being awake and content over the 24-hour period preceding admission. The Barr Chart has been formally validated in Australia against 24-hour voice-activated audiotapes of infant crying and fussing with which it is highly correlated [11].

#### Mental health status

*Edinburgh Postnatal Depression Scale *(EPDS) [12] is a 10-item self-rating scale used to screen for probable depression during the postnatal year in research and health care settings. It is acceptable to women and has high specificity and sensitivity in formal validation against diagnostic interviews [13]. The *Profile of Mood States *[14] is a 64-item mood adjective checklist that yields subscale scores on six dimensions of mood: anxiety, depression, irritability, fatigue, confusion, and functional efficiency.

### Analysis

The primary maternal outcome was maternal mood, assessed by mean EPDS and POMS subscale scores, coded as continuous variables, and proportion of scores in the clinical range coded as binary variables. The infant behaviour outcomes were maternal reports of the total number of hours the baby cried and fussed in 24 hours, coded as a continuous variable, and infant waking more than twice between 10 pm and 6 am, average daytime sleep two hours or less, and perceived poor overall quality of infant sleep, all coded as binary variables.

Maternal personality style (VPSQ) and intimate partner relationship (IBM), were analysed as continuous variables. Binary variables were created for the following maternal and infant factors. Maternal employment and education: categorised as professional/semi professional or other and post-secondary education versus other respectively; psychiatric history: having a past psychiatric diagnosis or not; history of reproductive difficulties: experience one or more adverse events in either a past or the most recent pregnancy or not; exposure to abuse or violence: a composite variable was derived from two items recording exposure to childhood abuse and/or violence in the past year and coded as exposure to one or both versus neither; maternal physical health: experiencing at least one health problem or not; coincidental adverse life events: experience of one or more adverse events or not; family support: receiving adequate help or not; capacity to confide in partner: always versus sometimes or not at all. Infant factors: feeding difficulties, colic, reflux, waking more than twice per night, total daytime sleep two hours or less and total length of infant crying in 24 hours more than 90 minutes were coded as binary variables.

Characteristics of study participants were summarised and compared to characteristics of all admissions of women with babies under 12 months to the service during the study period, and to Victorian data where available. Characteristics of infant behaviour on admission were summarised. Results are presented as means (standard deviation) and proportions for individual variables. The number of observations for each variable is presented with summary data. Characteristics of participants who provided complete data at the three assessment points were compared with those who were lost to follow-up. Chi square and t tests were used for univariate comparisons of dichotomous and continuous variables respectively. Known individual risk factors for poor maternal mental health were entered into multivariable regression to assess independent associations with EPDS score during admission using complete case analysis. Repeated measures analysis was used to assess change in outcomes over time. Data were analysed in SPSS v 11 [15].

## Results

### Recruitment and retention

Overall 153 mothers with babies under the age of 12 months were admitted during the study period (excluding women who had been admitted to the service under Protective Services provisions). Of these, seven had insufficient English to complete questionnaires, leaving 146 eligible participants. Of the 127 questionnaires that were distributed, 79 were completed and returned prior to participant's discharge (62%). At Time 2, 66/79 (84%) participants were retained, and at time 3, 58/79 (73%) participants remained in the study. Mean EPDS scores during admission of those who provided complete data and those who were lost to follow-up were similar (p > .05), but 5/13 women lost to follow-up (39 per cent) had reported intimate partner violence or coercion at Time 1, compared with 7/66 (11%) of women retained in the study, (χ^2^_1 _= 7.35, p = .017).

We describe the characteristics of study participants and their infants at admission and follow-up at one and six months after discharge, and the model describing factors associated with maternal mental health on admission to the service.

### Sociodemographic characteristics of the sample

Compared with all women admitted to the residential program during the study period with a baby under one year of age, and to all women who gave birth in Victoria in 2002 [16], study participants were broadly similar but somewhat more likely to be single and Australian born (Table 1).

**Table 1:** Characteristics of study participants

Characteristic	All admissions with a baby under 12 months during study period n = 153	Study sample n = 79	Victoria 2002*
**Maternal age mean (sd) years**	32.2 (5.1)	32.2 (4.9) range 18 to 43	30.2

**Infant age mean (sd) weeks**	33 (14.8)	31.0 (11.7) range 5 to 52	-

**Married/defacto n (%)**	133 (87)	68 (86)	87.3

**Single/separated/widowed n (%)**	17 (11)	11 (14)	11.4

**Australian born n (%)**	118 (77)	67 (85)	76.6

**Aboriginal & Torres Strait Islander n (%)**	1 (1)	0 (0)	0.7

* [16]

A high proportion of participants had post-secondary education qualifications (67%) and were in professional or semi-professional occupations (51%). Almost two thirds (49; 62%) of the sample were providing full-time primary care to their child(ren); 21 (27%) were in part time employment and 3 (4%) were employed full-time. Of the partners, 18 (14%) were either students or unemployed.

### Psychiatric history

A total of 25/77 (33%) reported a personal history of mental health problems, including distress requiring counselling (n = 20; 26%), depression (n = 6; 8%), eating disorder (n = 5, 7%), alcohol or drug dependence (n = 5; 7%). Overall, 14 (18%) reported a history of only one condition; 9 (12%) women reported two conditions and two women reported three co-morbid conditions.

### Reproductive history

Of the participants who had had more than one pregnancy (n = 51, 64.5%) three women (6%) reported a termination of pregnancy for fetal abnormality, 4 (8%) had given birth to a baby with a major abnormality, and three women (6%) had experienced one or more stillbirths. Twenty one women (27%) regarded the most recent conception as having been unplanned, only 49 (62%) were very pleased to have been pregnant and 4 (5%) per cent would rather the pregnancy had not happened at all. Conception had been assisted by IVF in 5 women (6%), and four (5%) had given birth to twins. The average gestation of babies born to the mothers in the sample was 39.4 weeks (range 32 - 42 weeks). Approximately half of the participants (44; 56%) had spontaneous vaginal births, 19 (24%) had instrumentally assisted vaginal births, 11 (14%) gave birth by caesarean section with a period of labour and 5 (6%) by caesarean section without labour, a total caesarean birth proportion of 20%.

### Experiences of childhood abuse and violence

One quarter (19/77; 25%) of women reported having been physically abused in childhood, almost as many (17; 22%) reported experiences of sexual abuse during childhood and 8 (10%) reported experiences of both. Thirteen (17%) women disclosed that they lived in current fear of their partner and/or had been hit, slapped, kicked or punched by their partner in the previous 12 months.

### Maternal physical health

Most women (66; 83%) had one or more current significant health problems including backache (43; 55%); headaches or migraine (28; 36%), problems with bowel control (20; 26%), haemorrhoids (17; 21%), unhealed or infected caesarean scar or episiotomy (15; 19%), urinary incontinence (14; 18%), or breast problems (7; 9%). Small numbers were taking prescribed medication, including 6 (8%) taking antidepressants.

### Coincidental life events

Almost half of the participants (36/78; 46%) reported that they were experiencing at least one coincidental distressing life event, including divorce proceedings, a child's health problem or disability, financial difficulties, unemployment and/or the need to return to employment, estrangement from family, and illness or death of family or friend.

### Family support

For many of the 79 participants, the grandparent generation was either unavailable or unable to assist; 19 (24%) of participants' mothers and 22 (28%) of their fathers were either unwell or deceased and a further 19 (24%) were divorced. Only 56 (71%) reported that their own mothers were delighted and enthusiastic about the birth of this baby, and 46 (58%) described their partners' mothers as responding in this way. A substantial proportion (35/79; 44%) reported that they were receiving insufficient practical and emotional support in the work of mothering, only 6 (8%) that other people were providing a lot of support and nearly half (35; 44%) wanted more help with household work and infant care, or support and education for parenting. More than half (26; 33%) reported feeling often or sometimes lonely and one third (26; 33%) felt in need of companionship.

### Intimate partner relationship

Of the 68 partnered women, very few (6; 9%) reported that their relationship with their partner had improved since the birth of the baby and more than half (40; 59%) had not attempted to have sex or were disappointed in their sexual relationship since the birth. Only 37 (54%) always felt able to confide in their partner. Mean (SD) Care subscale score, assessing partners as warm, considerate and affectionate, was 26.7 (7.4) and similar to the female population norm (27.1 (8.3); mean (SD) Control subscale score, assessing a relationship as critical and dictatorial, was 7.9 (6.9), somewhat better than the population norm (9.6 (8.3)) [7].

### Infant health and behaviour

The mean (sd) age of infants on admission was 31 (11.7) weeks. Almost two thirds (48; 61%) were having some breastfeeds. Reported feeding problems included refusal of breast, bottle or solid food and frequent small feeds. Many infants were sleeping little or not at all during the day and waking frequently at night (Table 2). There was wide variation in the reported total amount of infant crying in a 24 hour period; the mean was 1.7 hours and times ranged from none at all up to six and a half hours.

**Table 2:** Infant characteristics and behaviour during admission (n = 79)

Infant characteristic	n (%)
**Female**	33 (42%)

**Gestation < 37 weeks**	5 (6%)

**Feeding difficulties**	10 (13%)

**Diagnosed with colic or reflux prior to admission**	21 (27%)

**Taking medication for colic or reflux**	8 (10%)

**Waking at least 3 times between 10 pm and 6 am**	60 (76%)

**Having 2 hours or less daytime sleep**	60 (76%)

**Cries more than a little**	63 (80%)

**Cries for episodes of 10 minutes or more**	18 (23%)

Normal patterns of crying and sleep in the first twelve months of life vary greatly, but these data confirm that maternal reports of infant crying and sleep problems in this service exceed those reported in community samples, and that the two problems often co-occur [17,18] Despite these difficulties, on admission to the service most mothers (67; 85%) regarded themselves as fairly or very confident in their capacity to care for their infant.

### Maternal mental health during admission

The mean (SD) EPDS scores of participants was 11.3 (5.7), range 0 to 25, and more than one third (30; 39%) had scores in the clinical range (>12), indicating probable depression. This is significantly worse (p < 0.001) than reported in two large community samples in the UK and Australia assessed at 8 months postpartum (mean (SD) = 6.1 (5.79); 16.6% >12 [19] and mean (SD) = 7.2 (5.21); 8.8% >12 [20]. More than two thirds (55; 69%) scored in the clinical range for the POMS Fatigue-Inertia subscale indicating severe occupational fatigue.

### Factors associated with maternal mental health at admission

Risk factors for poor mental health and measures of difficult infant behaviours were entered into a linear regression model (Additional file 1). Women who are unassertive and sensitive to approval from others and who are unable to confide in their partners have significantly worse mental health. Worse mental health tended to be associated with experience of adverse coincidental life events, perceptions of partner behaviour as critical and controlling and frequent infant night waking. This model accounted for 28% of the variance in EPDS scores at admission (adjusted R square = 0.278).

### Evaluation of the residential program

On the second questionnaire one month after discharge, 66 (84%) of participants completed questions about the residential program. The majority agreed that they were very satisfied with the help (55; 85%), education (46; 70%) and support (55; 83%) they had received during admission to the residential program, and their involvement in planning their individual care (55; 83%). The support of nurses and the social interaction with other mothers were particular strengths of the program (Table 3).

**Table 3:** Evaluation of residential early parenting program (n = 66)

Aspect of program n (%)	Not helpful	Somewhat helpful	Very helpful
**Support of nurses**	1 (2)	7 (11)	58 (88)

**Education sessions**	6 (9)	38 (57)	22 (34)

**Staff presence while learning to settle baby**	2 (3)	22 (34)	42 (63)

**Assessment of baby**	3 (5)	18 (28)	44 (67)

**Sharing experience with other mothers**	1 (2)	16 (24)	50 (75)

### Changes in maternal mood at follow up

Between admission to the program and follow-up at one month, there were significant improvements on all measures of maternal psychological functioning, which were sustained at the six month follow-up assessment (Table 4).

**Table 4:** Maternal psychological functioning during admission and at follow-up

Measure	Admission Mean (sd)	1 month Mean (sd)	6 months Mean (sd)
**POMS sub-scale**			

**anxiety (n = 51)**	13.27 (6.92)	7.43 (5.97)**	6.88 (5.72)

**irritability (n = 49)**	11.43 (8.60)	7.00 (7.05) **	7.31 (6.77)

**depression (n = 50)**	16.34 (12.25)	7.78 (9.61) **	7.14 (9.48)

**clarity of thinking (n = 51)**	11.86 (6.34)	6.67 (4.80) **	6.06 (5.12)

**fatigue (n = 52)**	17.23 (6.37)	12.77 (6.75) **	11.85 (6.46)

**functional efficiency (n = 52)**	10.02 (6.13)	14.27 (7.00) **	15.04 (7.02)

**EPDS (n = 54)**	10.96 (5.33)	6.78 (4.44) **	6.26 (5.59)

** significant differences between admission and one-month follow-up (p ≤ 0.001)

POMS = Profile of Mood States

EPDS = Edinburgh Postnatal Depression Scale

This is also apparent in the proportions of women scoring in the clinical range on measures of psychological functioning during admission and at one and six month follow-up (Table 5). These indicate that one month after completing the program women are feeling less worried, sad and irritable and that their levels of energy, ability to think clearly and functional efficiency have improved. A sizeable group are still clinically exhausted in the months following admission.

**Table 5:** Scores in the clinical range on measures of psychological functioning (n; per cent)

	Admission (n = 77)	1 month (n = 61)	6 months (n = 58)
**POMS anxiety (>19)**	15 (20%)	5 (8%)	4 (7%)

**POMS depression (>23)**	14 (18%)	8 (13%)	4 (7%)

**POMS irritability (>13)**	24 (31%)	14 (23%)	10 (18%)

**POMS fatigue (>12)**	53 (69%)	26 (43%)	20 (35%)

**POMS confusion (>13)**	26 (34%)	9 (15%)	4 (7%)

**POMS vigour (<11)**	38 (49%)	20 (33%)	14 (25%)

**EPDS >12**	31 (39%)	11 (18%)	7 (12%)

The mean (SD) EPDS score for those in the probably depressed group during admission (EPDS > 12, n = 30) had declined from 17.1 (3.5) to 10.6 (5.6) by one-month post discharge (p < 0.001) and was 8.8 (5.9) at 6 months (ns). However there as a small group (n = 11) who continued to report depressive symptoms of sufficient severity to indicate probable depression at the one month follow-up assessment. High EPDS score at admission is associated with significantly greater likelihood (OR (95%CI) = 1.35 (1.02 to 1.79)) of being in the group who had not recovered at the follow-up assessment.

### Changes in infant behaviour

Similar improvements were reported in infant behaviour after discharge. The total length of crying and fussing in 24 hours had reduced from a mean (SD) of 101.02 (100.8) minutes during admission to 37. 7 (55.2) minutes at one month (p < 0.001) and 23.8 (30.1) minutes by six months post discharge (ns). Infants were sleeping longer during the day, with less frequent night-time waking. For most, these improvements were sustained six months post discharge (Table 6). These changes occurred regardless of infant age, and some may be attributable simply to developmental behavioural change.

**Table 6:** Changes in behaviour sleep (n; per cent)

	Admission (n = 79)	1 month (n = 66)	6 months (n = 58)
**Overall infant sleep poor or very poor**	61 (77%)	19 (29%)	9 (16%)

**Waking > twice per night**	60 (76%)	30 (45%)	19 (32%)

**Total daytime infant sleep ≤ 2 hours**	60 (76%)	25 (38%)	24 (42%)

Self-rated maternal confidence also improved over time. Almost all mothers (74; 94%) described themselves as fairly or very confident, which had increased to 96% at six months post-discharge.

## Discussion

This study adds to the growing body of evidence about Australia's residential early parenting services. A consecutive cohort of women was recruited, comprehensive self-report questionnaires were used to assess known risk factors for postpartum psychological disturbance, and the outcomes of interest were assessed with standardised psychometric measures. Although the sample was small, it was broadly representative of the population of women admitted to the service with a child under one year of age. However, the findings cannot be generalised to women of culturally and linguistically diverse backgrounds. As no screening measure of psychological state was used routinely at the service, the mental health of participants and non-participants could not be compared. Interpretation of these longitudinal data is hampered by the lack of a group of women with similar distress who did not participate in the residential program with which to compare the findings. The greater loss to follow-up of women with more complex problems, which were possibly less amenable to improvement through a brief intervention, may have led to a potential over-estimation of the effects of the program.

Overall, participants reported a high level of satisfaction with most aspects of the TCFHS residential program. Satisfaction surveys which are conducted by agencies external to the service are more reliable indicators of the views of respondents than those conducted in-house and these findings suggest that the content and format of group education sessions warrants review.

These findings confirm that mothers commonly present to residential early parenting services with infants who cry inconsolably for long periods, resist soothing and have seriously dysregulated sleep and feeding patterns. Frequently they have their own coexisting physical health problems, anxiety, depression, clinical exhaustion and adjustment difficulties [21-29]. Feeding, crying and sleeping problems are known to vary by infant age and developmental stage [17], but the sample size did not permit us to confirm this in this study.

Many women admitted to the brief residential programs at TCFHS also had difficult personal and social circumstances, including inadequate practical and emotional support from their family of origin and coincidental demanding life events. Some had a past personal history of psychiatric illness, and high rates of rare previous perinatal losses, assisted conception and operative interventions in childbirth. Persistent physical health problems were common and it is of concern that so much morbidity in mothers of infants remains untreated [30]. Although the small sample size limits precision of the estimate, the number of babies conceived by IVF in this sample (5; 6%) is four times higher than the national rate of births after assisted reproductive technology. This supports previous findings that ART is associated with elevated risk of early parenting difficulties [31,32].

The relationship with their partners was problematic for many participants, including an inability to confide, and in some, fear of intimidation or actual violence. Intimate partner violence is a known risk factor for depression and there has been under-ascertainment in postpartum populations [33,34]. The prevalence in this sample would suggest that it should be enquired about routinely, so that appropriate support and referral can be given. This contribution of the absence of a confiding relationship with the intimate partner to poor mental health on admission (Additional file 1) prompted a recent service innovation involving partners in a specific group-based team-parenting program which aims to assist couples to adjust roles and responsibilities in their primary relationship after the birth of a baby.

The numbers of infants who had been diagnosed and/or medicated for colic and reflux confirms that suspected gastro-oesophageal disease is common in infants admitted to residential early parenting services [35]. This is of concern given that there is an identifiable organic cause in only a small minority of babies with prolonged crying [36]. The poor quality of infant sleep at admission may have been causing infant exhaustion, with associated irritability and difficulties in settling, which are likely to make caring for a baby difficult and unrewarding [37]. When this persists for long periods, disabling occupational fatigue and emotional distress are likely. This represents a plausible mechanism for the well-documented relationship between dysregulated infant behaviour and maternal emotional distress, and a rationale for treating them together.

Parents play a central role in regulation of infant states [38]. A history of childhood abuse and violence is known to be associated with difficulties in emotional regulation and therefore in infant care and the quality of parent-infant attachment relationships [39]. We did not conduct formal assessments because of resource constraints and the wish to disrupt the program as little as possible in the conduct of the research [22] and therefore we can only speculate about the extent of parent-infant attachment problems in this cohort. However, a recent study conducted in a similar Victorian residential early parenting centre assessed parenting behaviour using the NCAST Parent Child Interaction (PCI) Teaching Scale [40] in a sample of 42 admitted mothers. They showed that when sociodemographic factors were adjusted for, mothers reporting that the pregnancy was unplanned, whose infant was born prematurely and who described their own sleep quality as poor were observed to be significantly less sensitive to their child's cues than other women [41]. Attachment theory provides a useful theoretical explanation for some of the crying and settling problems observed in our study and successful attachment-based therapies exist [42], suggesting a promising avenue for further research and practice review in this service. Consistent with the existing treatment approach in this service, it is also theoretically plausible that prolonged dysregulated infant behaviour which is not responsive to parents' attempts to soothe is anxiety-provoking and leads to hypervigilant parenting, overstimulated, tired infants and maternal exhaustion (Rowe H, Fisher J: Development of a universal psycho-educational intervention to prevent common postpartum mental disorders in primiparous mothers: a multiple method approach, submitted). Treatments that assist parents to learn sustainable infant sleep and settling strategies improve maternal self-efficacy, sleep quality and mood, and therefore strengthen parent-infant relationships.

Cross-sectional comparisons have found that mothers who are depressed are significantly more likely to report excessive infant crying and disturbed infant sleep and feeding than mothers who are not depressed [24,43,44]. The causal direction has been debated but Lam [45] demonstrated in a longitudinal follow-up of mothers and young children, that infant sleep problems preceded maternal depression. Residential parenting services, which are not psychiatrically designated, including at TCFHS, provide programs that include concurrent treatments to improve infant behaviour and manageability and maternal psychological functioning, and to strengthen parent-infant relationships [46].

Mild to moderate maternal depression, anxiety and clinical fatigue are responsive to non-pharmacological, psychological and psycho-educational interventions [4] and there is consistent evidence that infant behaviour management strategies have few risks and are effective in reducing frequent overnight waking, prolonging daytime sleep and reducing crying and fussing in infants with dysregulated sleep behaviour [47]. Effective early interventions for unsettled infant behaviour can prevent the development of later behavioural and emotional problems in children and adolescents [48]. Hiscock et al [49] showed in a randomised controlled trial that treatment of infant sleep disturbance resulted in marked improvements in maternal mood.

The findings of this study confirm the evidence from other uncontrolled prospective cohorts of the substantial, sustained improvements in maternal mental health and infant behaviour shown after discharge from Australian residential early parenting services [23,29,35,37,50-52], and greater and more rapid than those found in a sample treated only with infant settling strategies as outpatients [36,53]. Prospective cohort studies of this kind do not preclude the possibility that the improvements may have occurred because of the passage of time and increasing infant maturity. However, the results provide vital information on the presenting characteristics of women and infants on which to tailor treatment interventions, and are a necessary pre-requisite to a randomised controlled trial of treatment effectiveness.

There is a group of women for whom this intervention has been insufficient: six months after completing the program one in eight women still had significant psychological disturbance. In response to the results of this study, the service has introduced routine completion during admission of the EPDS. Women scoring above 12 on the EPDS are referred to the psychologist employed by the service. This is part of the introduction of a multidisciplinary model of care, which now also includes a social worker.

The associations between maternal depression and poor adherence to child health promotion and prevention measures including vaccination, attendance at well-child visits, use of recommended back-sleeping position and optimal breastfeeding [54] as well as poor child social and cognitive development [55] are well documented. Australia's residential early parenting programs address maternal mental health in the context of treatments for unsettled infant behaviour and education about child health and development, offering a model of integrated health care for families. Although we did not formally assess stigma in this study, admission to programs with structured content offered as part of residential early parenting services is likely to be less stigmatizing than psychiatric admission, to assist women to function within mainstream society, improve child development and family functioning, and to have benefits in a comprehensive system of mental health care for women with infants.

As part of Australia's perinatal depression initiative [56] the Australian Government 2008 budget allocated funding to state and territory governments to contribute to increased support and treatment services for women suffering from postnatal depression. The service evaluated in this study targets the whole family [46] and appears to provide a successful intervention for the majority of women who are admitted. This suggests that Victoria's early parenting services constitute an important component of the state's mental health support and treatment services for women. A comprehensive, integrated mental health care system will have good links between primary, secondary and tertiary services. Most women who self-referred to the service were already in the care of a maternal and child health nurse and/or a general practitioner. The unmet needs of the group for whom the intervention was insufficient suggest that a clear stepped pathway through care, underpinned by strong formal links to psychiatric treatment is essential [46].

## Conclusions

The focus of the TCFHS residential program is on parenting skills and modification of infant behaviour. The psychosocial circumstances and poor mental and physical health of the mothers constitute complex presenting problems and warrant comprehensive assessment and tailored treatment. Maternal mood and infant behaviour improved significantly after the completion of the program, adding to the evidence that this approach is an effective and acceptable early intervention for parenting difficulties and maternal mood disturbance. Service improvement could be achieved through additional targeted strategies to address more severe mood disturbance, women's physical health problems and the partner relationship. These findings suggest that residential early parenting services constitute and important part of a comprehensive mental health system for mothers of infants.

## Competing interests

The authors declare that they have no competing interests.

## Authors' contributions

JF initiated the study, contributed to design, development of data collection instruments, analysis and reporting. HR conducted the study, analysed the results and drafted the paper. Both authors read and approved the final manuscript.

## Supplementary Material

Additional file 1 **Factors associated with maternal mental health status (EPDS score) (Additional file**1**Rowe and Fisher Table 3.doc).** Click here for file
