# Supplementary material for: The contribution of Australian residential early parenting centres to comprehensive mental health care for mothers of infants: evidence from a prospective study
Source: Int J Ment Health Syst. 2010 Apr 11;4:6. doi: 10.1186/1752-4458-4-6 (PMC2873569; doi:10.1186/1752-4458-4-6)
Supplement: Additional file 1 — Factors associated with maternal mental health status (EPDS score) (Additional file1Rowe and Fisher Table 3.doc). [file 1752-4458-4-6-S1.DOC]

## Table 3 Factors associated with maternal mental health status (EPDS score)

| **Factor** | **n (%)** | **Mean EPDS score** | **p for difference** | **Standardised β coefficient (95%CI)** | **p** |
| --- | --- | --- | --- | --- | --- |
| **Maternal history** | | | | | |
| **Adverse psychiatric history (n=76)**  **No**  **Yes** | 63 (83%)  13 (17%) | 10.6  13.7 | 0.07 | -0.03 (-4.38, 3.46) | 0.82 |
| **VPSQ Vulnerability subscale (n=77)** | Pearson correlation =0.424; |  | p<0.001 | 0.37 (0.15, 0.76) | 0.004 |
| **Adverse reproductive history (n=77)**  **No**  **Yes** | 35 (47%)  42 (53%) | 12.2  10.5 | 0.18 | -0.18 (-4.52, 0.66) | 0.14 |
| **Exposure to abuse or violence (n=66)**  **No**  **Yes** | 39 (61%)  27 (39%) | 11.2  10.8 | 0.77 | 0.008 (-2.52 to 2.70) | 0.95 |
| **Health and social circumstances** | | | | | |
| **At least one physical health problem (n=77)**  **No**  **Yes** | 13 (17%)  64 (83%) | 10.7  11.4 | 0.70 | -0.09 (-4.08, 1.87) | 0.46 |
| **Coincidental adverse life events (n=77)**  **No**  **Yes** | 41 (54%)  36 (46%) | 10.1  12.6 | 0.06 | 0.22 (-0.29, 5.01) | 0.08 |
| **Needs extra help and support (n=77)**  **No**  **Yes** | 29 (37%)  48 (63%) | 11.0  11.4 | 0.76 | 0.03 (-2.94, 2.34) | 0.82 |
| **Partner factors** | | | | | |
| **IBM Care subscale (n=72)** | Pearson correlation = -0.116; |  | 0.33 | 0.20 (-0.08 to 0.38) | 0.19 |
| **IBM Control subscale (n=72)** | Pearson correlation =0.118; |  | 0.32 | 0.23 (-0.03 to 0.38) | 0.09 |
| **Always able to confide in partner**  **(n=68; n(no partner) =9)**  **Yes**  **No** | 37 (54%)  31 (46%) | 9.5  12.9 | 0.01 | 0.29 (0.48, 5.67) | 0.02 |
| **Infant factors** | | | | | |
| **Infant night waking at least twice (n=76)**  **No**  **Yes** | 17 (23%)  59 (77%) | 10.0  11.6 | 0.31 | 0.19 (-0.61, 5.56) | 0.11 |
| **Infant crying in 24 hours > 90 mins (n=69)**  **No**  **Yes** | 43 (62%)  26 (38%) | 10.3  12.4 | 0.13 | -0.01 (-2.85, 2.56) | 0.92 |

EPDS Edinburgh Postnatal Depression Scale (12)

VPSQ Vulnerable Personality Style Questionnaire (8)

IBM Intimate Bonds Measure (7)
